# Childhood behaviours and adverse economic and social outcomes – can we improve detection and prevention?

**DOI:** 10.1192/j.eurpsy.2021.249

**Published:** 2021-08-13

**Authors:** F. Vergunst, R. Tremblay, D. Nagin, F. Vitaro, S. Cote

**Affiliations:** 1 Public Health, University of Montreal, Montréal, Canada; 2 Psychoeducation, Umiversity of Montreal, Montreal, Canada; 3 Statistics, Carnegie Mellon University, Pitsburgh, United States of America

**Keywords:** ADHD, Conduct disorder, Screening, prevention

## Abstract

**Introduction:**

Disruptive behaviours underpin the most prevalent and costly psychiatric disorders in youth including ADHD and conduct disorder. Yet the association between childhood behavioural problems and economic and social outcomes in adulthood are rarely examined in a population-based samples where early detection and prevention may be possible.

**Objectives:**

To examine the association childhood behavioural problems and economic and social outcomes from age 18-35 years across three studies.

**Methods:**

This study daws on 30-year Canadian birth cohort (n=3017) linked to government tax return records. Behavioural assessments – for inattention, hyperactivity, opposition, aggression, anxiety and prosociality – were prospectively obtained from teachers when children were aged 6-12 years. Regression models were used to link behavioural assessments in kindergarten (age 5/6 years) to earnings at age 33-35 years (Study 1) and to trajectories of welfare receipt (Study 2), while behaviour at age 10-12 years was linked to trajectories of partnering. Children’s IQ and family background were adjusted for.

**Results:**

Inattention, aggression-opposition (males only) and low low-prosociality in kindergarten were associated with lower earnings at age 33-35 years (Study 1), inattention, aggression-opposition and low prosociality in kindergarten predicted following a chronic welfare receipt trajectory from age 18-35 (Study 2), and inattention, aggression-opposition, anxiety and low-prosociality at age 10-12 years were associated with increased likelihood of being unpartnered and with partnership dissolution from age 18-35 years (Study3).

**Conclusions:**

Behavioural assessments made by schoolteachers can identify children at risk of adverse economic and social outcomes in adulthood. The implications of for early screening and prevention will be discussed.

**Disclosure:**

No significant relationships.

